# Identification of a Gene Panel Predictive of Triple-Negative Breast Cancer Response to Neoadjuvant Chemotherapy Employing Transcriptomic and Functional Validation

**DOI:** 10.3390/ijms231810901

**Published:** 2022-09-17

**Authors:** Radhakrishnan Vishnubalaji, Hikmat Abdel-Razeq, Salahddin Gehani, Omar M. E. Albagha, Nehad M. Alajez

**Affiliations:** 1 Translational Cancer and Immunity Center (TCIC), Qatar Biomedical Research Institute (QBRI), Hamad Bin Khalifa University (HBKU), Qatar Foundation (QF), Doha P.O. Box 34110, Qatar; 2 Department of Internal Medicine, King Hussein Cancer Center, Amman 11941, Jordan; 3 School of Medicine, The University of Jordan, Amman 11942, Jordan; 4 Hamad Medical Corporation, Doha P.O. Box 34110, Qatar; 5 College of Health & Life Sciences, Hamad Bin Khalifa University (HBKU), Qatar Foundation (QF), Doha P.O. Box 34110, Qatar; 6 Centre for Genomics and Experimental Medicine, Institute of Genetics and Cancer, University of Edinburgh, Edinburgh EH4 2XU, UK

**Keywords:** TNBC, neoadjuvant chemotherapy, predictive biomarkers, pathological complete response, residual disease

## Abstract

Triple-negative breast cancer (TNBC) patients exhibiting pathological complete response (pCR) have better clinical outcomes compared to those with residual disease (RD). Therefore, robust biomarkers that can predict pCR may help with triage and resource prioritization in patients with TNBC. Herein, we identified a gene panel predictive of RD and pCR in TNBC from the discovery (*n* = 90) treatment-naive tumor transcriptomic data. Eight RD-derived genes were identified as TNBC-essential genes, which were highly predicative of overall survival (OS) and relapse-free survival (RFS) in an additional cohort of basal breast cancer (*n* = 442). Mechanistically, targeted depletion of the eight genes reduced the proliferation potential of TNBC cell models, while most remarkable effects were for combined SLC39A7, TIMM13, BANF1, and MVD knockdown in conjunction with doxorubicin. Orthogonal partial least squares-discriminant analysis (OPLS-DA) and receiver operating characteristic curve (ROC) analyses revealed significant predictive power for the identified gene panels with an area under the curve (AUC) of 0.75 for the validation cohort (*n* = 50) to discriminate RD from pCR. Protein–Protein Interaction (PPI) network analysis of the pCR-derived gene signature identified an 87-immune gene signature highly predictive of pCR, which correlated with better OS, RFS, and distant-metastasis-free survival (DMFS) in an independent cohort of basal and, to a lesser extent, HER2+ breast cancer. Our data have identified gene signatures predicative of RD and pCR in TNBC with potential clinical implications.

## 1. Introduction

Triple-negative breast cancer (TNBC) represents 15–20% of all breast cancers, which is characterized by the lack of hormone receptors (HR), including estrogen receptor (ER) and progesterone receptor (PR) expression, in addition to the lack of epidermal growth factor receptor-2 (HER-2) amplification [1]. Despite the lower incidence of TNBC compared to other molecular subtypes, TNBC is oftentimes presented with an advanced tumor stage and those patients have worse overall survival (OS) [2]. Neoadjuvant chemotherapy (NAC) followed by surgery is the standard-of-care clinical practice for TNBC with larger tumors [3]. Longitudinal patterns of TNBC response to NAC are oftentimes used to assess treatment response and likelihood for OS. Pathological complete response (pCR) is associated with better prognosis, while residual disease (RD) predicts worse clinical outcome [4]. Therefore, the timely identification of patients who will not benefit from NAC is critical to avoid exposure of this patient group to toxic drugs and unnecessary delays in surgical intervention.

While tumor heterogeneity and clonal evolution have been linked to variable responses to NAC in TNBC employing single-cell analysis [5,6], currently there are no predictive signatures for RD and pCR in the context of NAC in clinical practice. We previously reported the presence of immune gene expression as the hallmark of pCR in TNBC employing single-cell analysis [7]. In the current study, we utilized publicly available datasets and characterized the transcriptome from a discovery cohort (*n* = 90) transcriptomic data and subsequently constructed an orthogonal partial least squares-discriminant analysis (OPLS-DA) and validated it on a second cohort (*n* = 50) of TNBC and identified the gene signatures predicative of RD and pCR. Interestingly, we observed genes associated with pCR to have better prediction (higher AUC) compared to genes associated with RD, which implied inherent heterogeneity in the genes driving RD, while the genes predictive of pCR were mostly indicative of immune infiltration. Employing CRISPR-Cas9 in vitro screen data, we identified eight RD-derived genes essential for TNBC, which upon targeted depletion reduced TNBC colony formation and enhanced their sensitivity to doxorubicin and paclitaxel. 

## 2. Results

### 2.1. Differential Expression Analysis of RD Compared to pCR TNBC Tumors 

**Figure 1:**
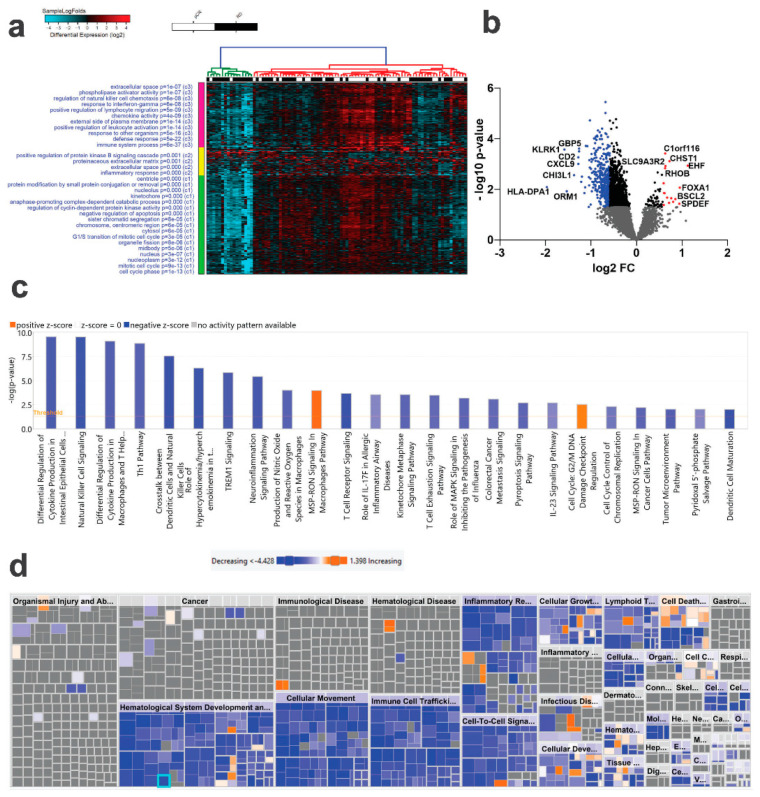
**Comparative analysis of mRNA expression in TNBC patients as a function of pathological complete response (pCR).** TNBC patients were grouped according to pCR (*n* = 38) and residual disease (RD, *n* = 62) and were subjected to differential analysis (1.5 FC, *p*< 0.05). (**a**) Hierarchical clustering of TNBC as a function of pCR (*n* = 38) or RD (*n* = 62) based on differentially expressed mRNA genes. Each column represents one patient sample, and each row represents an mRNA. The expression level of each gene (log2) in a single sample is shown according to the color scale. (**b**) Volcano plot illustrating the upregulated (red) or downregulated (blue) genes in RD vs. pCR TNBC. The most differentially expressed mRNAs are indicated on the plot. (**c**) IPA canonical pathway analysis on the differentially expressed genes in RD vs. pCR. (**d**) IPA disease and function analysis of differentially expressed genes in RD vs. pCR. Orange color indicate activation while blue color indicates suppression.

To identify putative markers predictive of RD and pCR in the context of NAC therapy for TNBC, we retrieved RNA-Seq data from 90 TNBC patients who exhibited variable responses to NAC (pCR; *n* = 38 and RD; *n* = 52). The data were mapped to the gencode release v33 followed by comparative analysis, which revealed several differentially expressed genes, with most of the genes exhibiting downregulated expression in RD compared to pCR (1.5 FC, *p* < 0.05, Figure 1a and Table S1). Differentially expressed genes in RD and pCR are illustrated as a volcano plot (Figure 1b), with upregulated genes in RD are shown in red and downregulated genes in blue. To gain deeper insight into functional categories associated with RD vs. pCR TNBC, the list of differentially expressed genes were subjected to ingenuity pathway analysis (IPA). Canonical pathway analysis on the differentially expressed genes revealed predominant under representation of categories associated with immune response (Figure 1c). Figure 1d provides a high-level tree map of altered downstream functional categories utilizing the list of differentially expressed genes in RD vs. pCR. The major-colored rectangles indicate a group of associated biological functions or diseases, where blue indicate decreasing and orange indicates increasing, and the dimension of the rectangles indicates where associated functions are predicted to be up or down, while the color intensity corresponds to the absolute Z-scores. Using this analysis, IPA revealed suppression of various immune functions as the hallmark associated with RD, implying activation of immune functions as the most significant surrogate marker to pCR in TNBC in the context of NAC response (Figure 1d and Table S2). 

### 2.2. Identification of mRNA Transcripts Predicative of RD and pCR 

**Figure 2:**
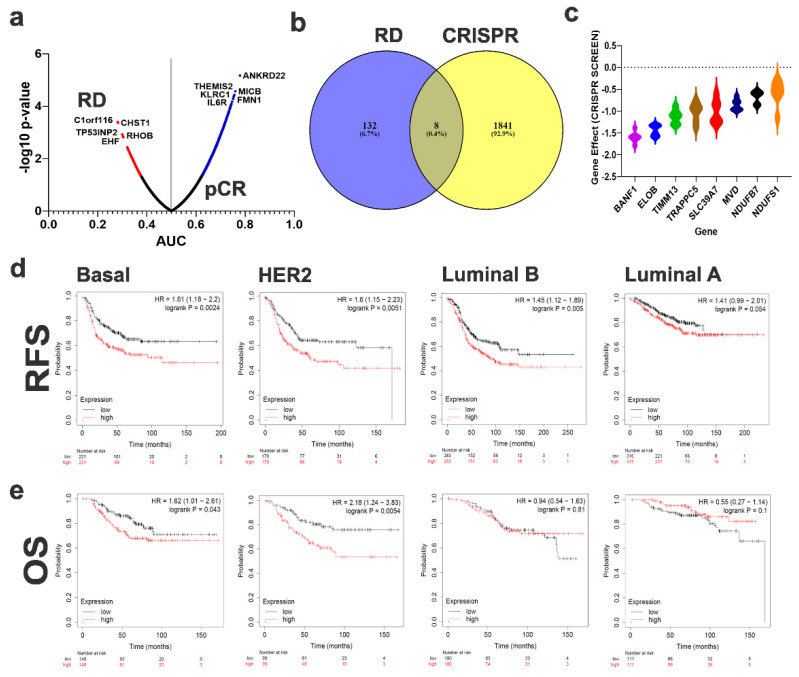
**Identification of predictive mRNA panels to discriminate RD from pCR TNBC.** (**a**) mRNA transcriptome data were subjected to ROC analysis. Data are presented as a volcano plot where the *x*-axis represent the AUC and the *y*-axis represent the −log10 *p*-value. mRNAs with significant (*p* < 0.05) prediction power of RD are labeled in blue, while those predictive of pCR are colored red. (**b**) Venn diagram representing the common genes between mRNAs predictive of RD from the discovery cohort and the gene effects (<−0.5) from a CRISPR-Cas9 screen on TNBC cell models from the Achille project. (**c**) Violin plot illustrating the gene effects of the eight identified mRNAs in a panel of TNBC cell models (BT549, MDA-MB-157, HCC70, MDA-MB-231, MDA-MB-468, MDA-MB-453) based on data from the Achille project. RFS (**d**) and OS (**e**) analysis of the eight gene signature in an independent cohort of Basal, HER2+, Luminal B, and Luminal A breast cancer patients.

Given the limited number of differentially expressed genes using differential analysis, we subsequently performed receiver operating characteristic (ROC) analysis to identify the set of genes which can predict the response of TNBC patients to NAC (RD vs. pCR). ROC analysis identified 140 genes predictive of RD and 1490 genes predictive of pCR (asymptotic *p*-value < 0.05) as illustrated in the volcano plot (Figure 2a and Table S3). C1orf116, CHST1, TP53INP2, RHOB, SLC9A3R2, EHF, BAD, FAM89B, PEBP1, and RHOC were the top 10 genes predictive of the RD phenotype. PPI network analysis of genes predictive of RD revealed a weak association (Figure S1). Therefore, we employed an alternative strategy to identify potential essential genes for the RD phenotype by crossing the enriched genes in the RD from ROC analysis with the CRISPR-Cas9 pooled library functional screen data from the Achilles project in TNBC [8]. Data presented in Figure 2b shows the overlap between the enriched genes in RD and genes with significant effects on TNBC survival (effect < −0.5), revealing eight common genes (ELOB, SLC39A7, TIMM13, BANF1, NDUFS1, NDUFB7, TRAPPC5, and MVD). The gene effect of the eight RD gene signature on various TNBC cell models based on the CRISPR-Cas9 screen is illustrated in Figure 2c as a violin plot, where a lower score indicates that the TNBC cell line is highly dependent on the corresponding gene for survival. The eight-gene RD signature was subsequently subjected to survival analysis using mean expression for the eight genes in each subject, which revealed significant predictive power for this signature on RFS (Figure 2d) and OS (Figure 2e) for basal and HER2+ breast cancer, but not for those with Luminal A and Luminal B tumors.

### 2.3. Targeted Depletion of RD-Essential Genes Inhibited TNBC Colony Forming Unit (CFU) Potential and Enhanced Sensitivity to NAC

**Figure 3:**
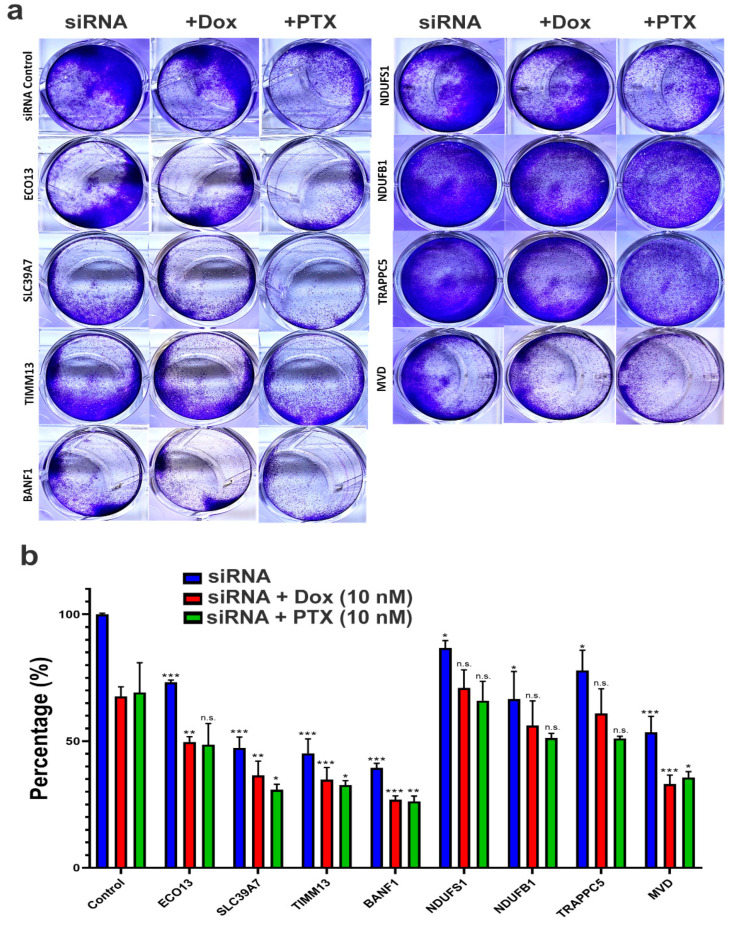
**Effects of RD-genes knockdown on MDA-MB-231 CFU and viability.** (**a**) Representative image showing CFU potential of MDA-MB-231 cells on day 7 post transfection with siRNA targeting ELOB, SLC39A7, TIMM13, BANF1, NDUFS1, NDUFB7, TRAPPC5, and MVD as a single treatment modality or in combination with doxorubicin (10 nM) or paclitaxel (10 nM). (**b**) Quantification of CFU from two independent experiments. Data are presented as the mean ± S.E., *n* = 6. * *p* < 0.05, ** *p* < 0.005, *** *p* < 0.0005, n.s.: not significant. Each treatment was compared to the corresponding control condition.

To validate the potential role of the identified eight genes in mediating RD to NAC, we used siRNA to suppress the expression of ELOB, SLC39A7, TIMM13, BANF1, NDUFS1, NDUFB7, TRAPPC5, and MVD in MDA-MB-231 cells and assessed their effects on CFU as single agent or in combination with doxorubicin and paclitaxel. Data presented in Figure 3a illustrated significant inhibition of CFU following gene knockdown, which was most remarkable in BANF1, TIMM13, SLC39A7, MVD, NDUFB1, ECO13, TRAPPC5, and NDUFS1, respectively. Interestingly, the effects were further enhanced when BANF1, MVD, TIMM13, SLC39A7, and ECO13 were combined with doxorubicin. Similarly, the effects were most remarkable when BANF1, SLC39A7, TIMM13, and MVD were combined with paclitaxel. Similar results were also obtained when using the BT-549 TNBC models (Figure S2). 

**Figure 4:**
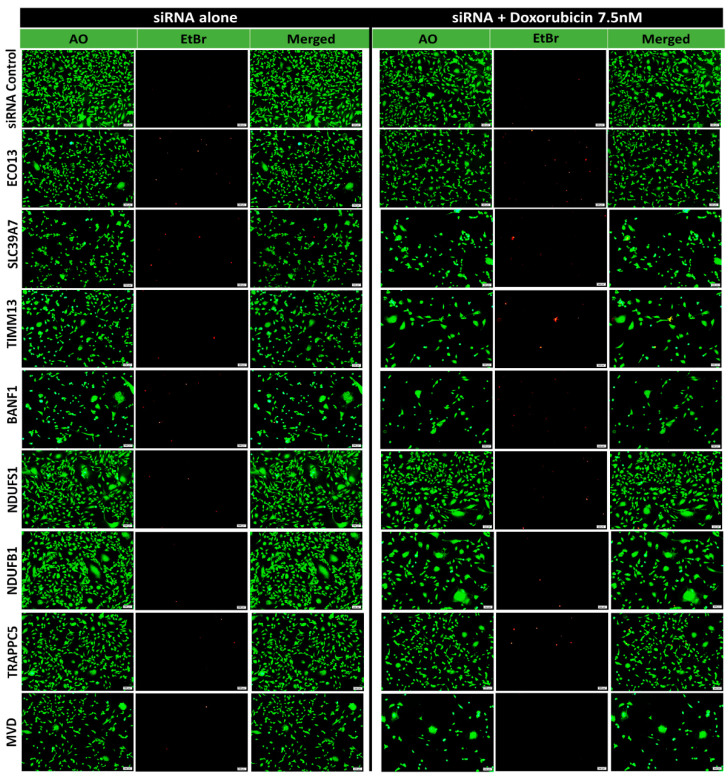
**Viability fluorescent microscopy of MDA-MB-231 cells in response to siRNA-mediated gene silencing.** MDA-MB-231 cells were transfected with the indicated siRNAs as single agent or in combination with doxorubicin (7.5 nM) and were visualized under fluorescent microscope for AO/EtBr staining on day 7. Representative images are shown.

The results were further confirmed using acridine orange and ethidium bromide viability staining in the MDA-MB-231 which revealed induction of cell death in siRNA knockdown cells and was further enhanced when combined with doxorubicin (Figure 4), therefore corroborating a role for these genes in TNBC survival and sensitive to doxorubicin.

### 2.4. Discriminant Analysis 

**Figure 5:**
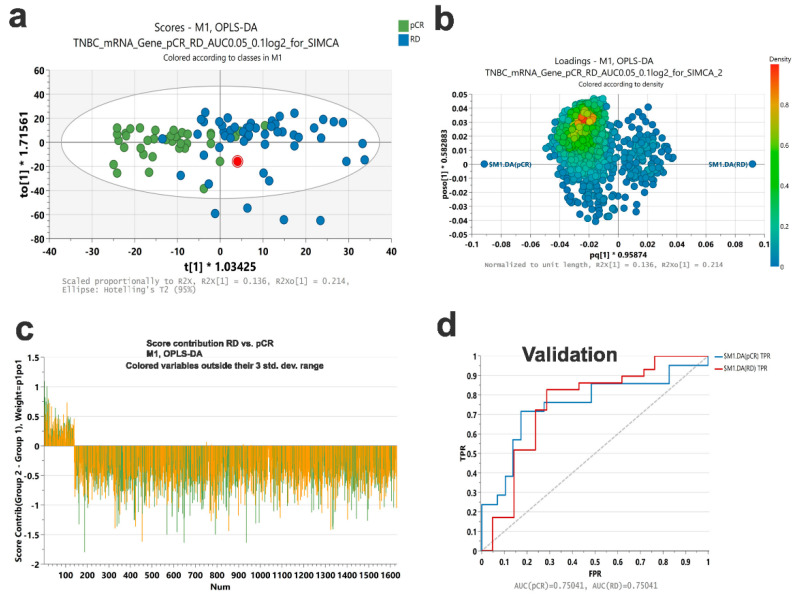
**Validation of a gene signature predictive of RD and pCR in TNBC.** (**a**) OPLS-DA score plot of a RD and pCR gene signature based on ROC analysis. (**b**) Loading plot based on SIMCA OPLS-DA analysis. Variables are colored according to their density score. (**c**) Score contribution plot illustrating the contribution of each variable in the model. (**d**) ROC analysis of the identified gene signature predictive of RD and pCR in an independent validation cohort consisting of 29 RD and 21 pCR. The *y*-axis represents FPR while the *x*-axis represents TPR.

To determine the potential predictive value of the identified genes, we performed Orthogonal Projections to Latent Structures Discriminant Analysis (OPLS-DA) on the discovery cohort. Data from the OPLS-DA model is presented as score plots to visualize the differences between RD and pCR (Figure 5a). The loading plot in Figure 5b indicates the variables that express the observed difference between RD and pCR, where each variable is colored according to density score. The score contribution of each variable is shown in Figure 5c. The variable influence on projection (VIP) predicted score for each variable is listed in Table S4. The model was tested on the same discovery cohort which revealed an AUC = 0.93 to discriminate RD from pCR (Figure S3) and was subsequently validated on a second cohort of TNBC (*n* = 50) revealing an AUC = 0.75 to discriminate RD from pCR in this independent validation cohort (Figure 5d). 

### 2.5. PPI Network Analysis for Genes Predictive of pCR

**Figure 6:**
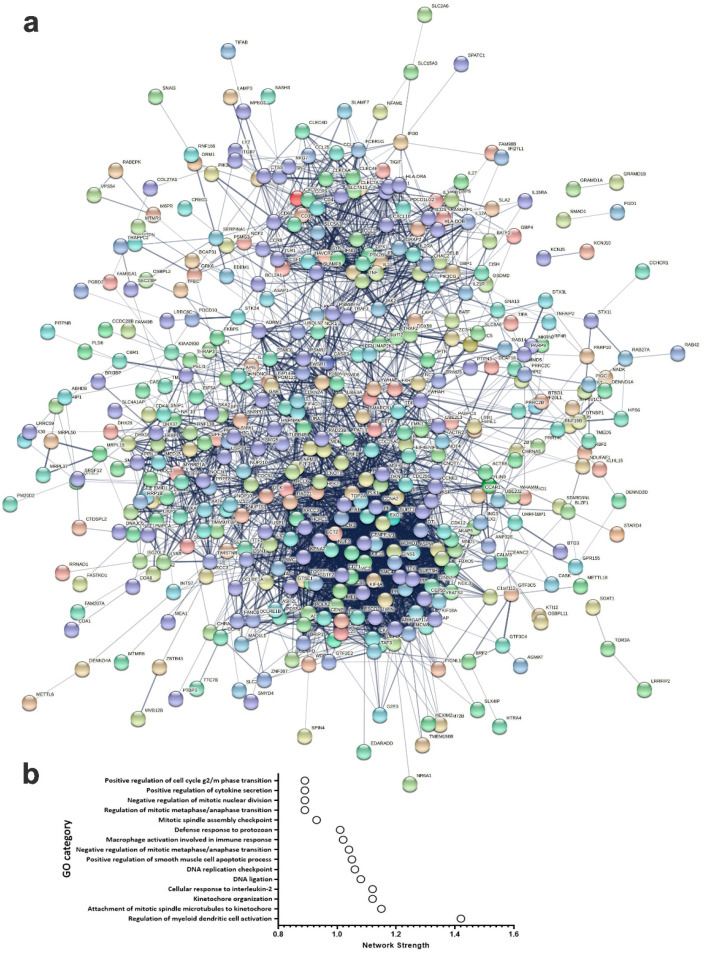
**PPI network analysis of mRNAs predictive of pCR.** (**a**) The 500 common mRNAs identified through ROC analysis predicative of pCR in the discovery and validation cohorts were subjected to PPI network analysis using the STRING database. Network statistics: number of nodes: 500; number of edges: 3256; average node degree: 13; avg. local clustering coefficient: 0.402; expected number of edges: 1631; PPI enrichment *p*-value: <1.0 × 10^−16^. (**b**) Bar graph illustrating the top 15 enriched gene ontology (GO) categories (*y*-axis) with network strength score presented on the *x*-axis.

**Figure 7:**
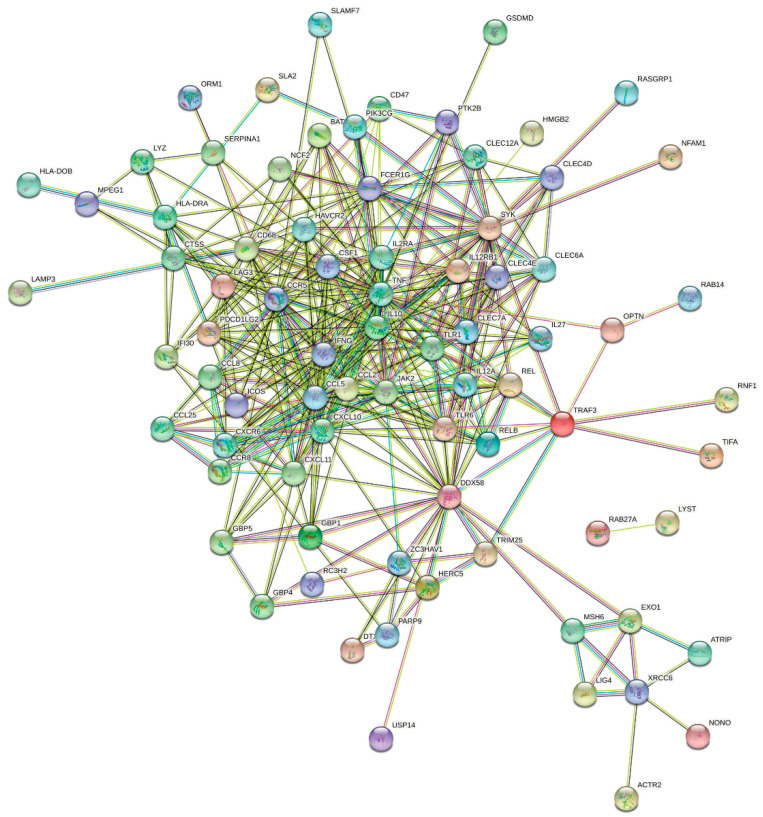
**PPI network analysis of an 87-immune-gene signature predictive of pCR.** Eighty-seven immune gene signature predictive of pCR were subjected to PPI network analysis using the STRING database. Network statistics: number of nodes: 87; number of edges: 403; average node degree: 9.26; avg. local clustering coefficient: 0.591; expected number of edges: 76; PPI enrichment *p*-value: <1.0 × 10^−16^.

In order to gain more insight in to the functional and network enrichment in pCR, the list of common genes predicative of pCR from the discovery and validation cohorts using univariate analysis were imported into the STRING database and were subjected to network analysis. Data presented in Figure 6a illustrates a dense network with multiple interactions. Interestingly, the highest network enrichment score was for functional categories related to immune and cell cycle regulation (Figure 6b and Table S5). Given the apparent role of immune signatures in predicting pCR, we identified an 87-immune signature base on GO analysis, which was subjected to separate STRING analysis revealing activation of various immune processes such as innate immunity, inflammatory response, myeloid leukocyte activation, adaptive immune response, activation of neutrophils and natural killer cells as the most affected functional categories (Figure 7 and Table S6). 

### 2.6. Survival Analysis of 87-Immune-Gene Signature in an Independent Breast Cancer Cohorts 

**Figure 8:**
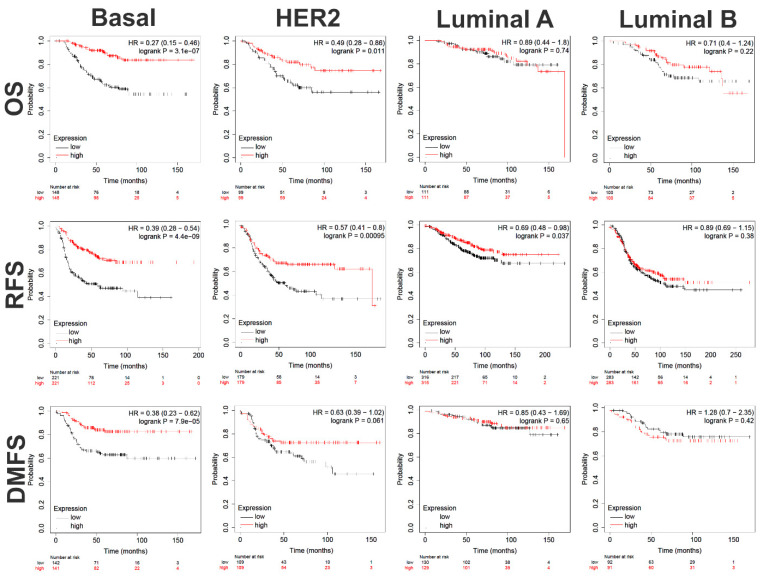
**Survival analysis of an 87-immune-gene signature in independent breast cancer cohorts.** The identified 87-immune-gene signature predictive of pCR was subjected to RFS, OS, and DMFS in a cohort of basal (RFS: *n* = 442, OS: *n* = 296, DMFS: *n* = 283), HER2+ (RFS: *n* = 358, OS: *n* = 198, DMFS: *n* = 218), Luminal B (RFS: *n* = 566, OS: *n* = 200, DMFS: *n* = 183), and Luminal A (RFS: *n* = 631, OS: *n* = 222, DMFS: *n* = 259).

The identified 87-immune-gene signature was assessed for association with OS, RFS, and DMFS in additional cohorts from the KMplotter database, a commonly used database for cancer survival analysis. Interestingly, the 87-immune-gene signature was highly predictive for better clinical outcome for OS (HR = 0.27 (0.15–0.46), logrank P = 3.1 × 10^−7^); RFS (HR = 0.39 (0.28–0.54), logrank P = 4.4 × 10^−9^); and DMFS (HR = 0.38 (0.23–0.62), logrank P = 7.9 × 10^−5^) in basal breast cancer (Figure 8, first panel). To a lesser extent, the signature also predicted better clinical outcomes for OS (HR = 0.49 (0.28–0.86), logrank P = 0.01), RFS (HR = 0.57 (0.41–0.8), logrank P = 0.0009), and DMFS (HR = 0.63 (0.39–1.02), logrank P = 0.06) in HER2+ breast cancer patients (Figure 8, second panel). The signature also predicted better RFS (HR = 0.69 (0.48–0.98), logrank P = 0.037) for Luminal A, but not for Luminal B as well as neither predicted OS nor DMFS for Luminal A nor Luminal B breast cancer patients, suggesting its selectivity in predicting Basal, and to a lesser extent, HER2+ breast cancer patient outcomes (Figure 8, third and fourth panels). 

## 3. Discussion

The quest to identify potential biomarkers predictive of TNBC response to NAC have been a daunting task, that nevertheless remains important to pursue for patient triage and resource prioritization. In the current study we took multiple approaches to define gene signatures predicative of TNBC pCR and RD and the potential targeting of RD-associated genes to enhance the efficacy of standard NAC. Our data identified 140 genes to be predicative of RD, while the bulk of differentially expressed genes were found to be associated with pCR. This finding implies that genes associated with RD are likely driven by tumor cells, with each patient having their own oncogenic signatures. On the other hand, the signature predictive of pCR was mostly driven by immune infiltrating cells; therefore, it shows higher predicative power and was largely present across patients from the discovery and validation cohorts. In our quest to identify potential RD-essential genes, we integrated our computational and prediction data with CRISPR-Cas9 functional screen data from the Achilles project, leading to the identification of eight essential genes for the RD phenotype. The most catastrophic effects of gene knockdown were for BAF Nuclear Assembly Factor 1 (BANF1), Translocase of Inner Mitochondrial Membrane 13 (TIMM13), Solute Carrier Family 39 Member 7 (SLC39A7), and Mevalonate Diphosphate Decarboxylase (MVD). A recent report suggested a correlation between elevated expression of BANF1 and lymph node metastasis status and TNM staging in TNBC [9]. In addition, BANF1 has been shown to regulate PARP1-directed DNA damage response to oxidative stress [10]. Thus far, TIMM13 has not been linked to cancer, so our data suggest a plausible role for this gene in TNBC response to NAC. Knockdown of SLC39A7 was show to inhibit cell growth and induce apoptosis in colorectal cancer (CRC) [11]. Recent data suggested SLC39A7 to promote glioma cell malignancy through the TNF-α-mediated NF-κB pathways [12]. Elevated expression of SLC39A7 was also reported in tamoxifen-resistant breast cancer cells [13]. However, our data are the first to implicate SLC39A7 in the response of TNBC to NAC. Among the identified RD genes, MVD has not been linked to human cancers thus far. Elongin B (ELOB) knockdown exhibited minimal effects on TNBC viability, which was significantly enhanced when combined with doxorubicin and paclitaxel. ELOB is one subunit of the transcription factor B complex. Although there is currently no report connecting ELOB with human cancers, our data suggest its potential use to enhance TNBC sensitivity to NAC. 

Interestingly, we observed the presence of an immune gene signature as a common feature in TNBC who responded to NAC in two independent cohorts. In particular, we identified an 87 gene signature that predicted better OS, RFS, and DMFS in basal and HER2+ breast cancer. Our data are consistent with recently published reports [14,15] and with our recently published work on single-cell analysis of TNBC in the context of TNBC response to NAC [7]. This interesting observation raises a key question about whether TNBC tumors with heavy immune infiltration represent different molecular subtype with different sensitivity to NAC compared to tumors lacking immune infiltration, which warrants further investigation. Alternatively, does the presence of immune infiltration enhance NAC efficacy? The identified 87 immune-gene signature included LAG3, RELB, CCL2, IFNG, MSH6, ZC3HAV1, CD68, ORM1, LYZ, USP14, SLA2, HERC5, LAMP3, NONO, BATF, FCER1G, CCR5, REL, DTX3L, HMGB2, C2, CLEC4E, CLEC4D, CLEC7A, IL12A, CXCL10, CXCL11, RASGRP1, HAVCR2, ICOS, ATRIP, TRIM25, RNF166, CCR8, CSF1, NFAM1, TUBB4B, LYAR, CLEC12A, IL27, PIK3CG, XRCC6, PARP9, DNAJC5, MPEG1, TIFA, TLR1, CD47, EXO1, NCF2, SLAMF7, CTSS, GBP5, GBP4, GBP1, CREG1, RNF19B, RC3H2, RAB14, SYK, ACTR2, KCNAB2, OPTN, DDX58, IL2RA, JAK2, CLEC6A, LYST, CCL25, CCL8, HLA-DRA, RAB27A, PTK2B, PDCD1LG2, IFI30, TLR6, DSN1, HLA-DOB, CXCR6, TNF, IL10, SERPINA1, GSDMD, TRAF3, IL12RB1, CCL5, and LIG4. Several of those genes were also identified in our previous single-cell analysis such as LYZ, CXCL10, CD47, SLAMF7, CTSS, GBP1, and HLA-DRA. Of particular interest, SLAMF7 has recently been shown to program T cells toward exhaustion phenotype via SLAMF7+ tumor-associated macrophages (TAMs) in the tumor microenvironment of renal cell carcinoma [16]. A recent study suggested expression of SLAMF7 to promote cytotoxicity of human NK cells [17]. IFNG was also among the identified genes predicative of pCR. Concordantly, our recent single-cell analysis also identified activation of the IFNG pathway in TNBC patients exhibiting pCR. Taken together, our data have highlighted activation of immune cells as the hallmark associated with pCR. 

One limitation of the current study is the relatively small sample size of the validation cohort. Nonetheless, only expression data were available from the validation cohort. Interestingly and despite the differences in bioinformatics tools and reference genome used for the discovery and validation cohorts, the gene signature showed reasonable performance in the validation cohort (AUC = 0.75). However, those findings remain to be validated in additional cohorts and in TNBC patients receiving other NAC regimens. 

Our results identified gene panels predictive of RD and pCR in TNBC, where the strongest prediction was for pCR. Eight genes were identified as TNBC-essential genes, which were highly predicative of OS and RFS, and were essential for TNBC proliferation. Interestingly, pCR-associated genes were enriched for immune cell functions and were predictive for better OS, RFS, and DMFS in basal and to a lesser extent in HER2+ breast cancer patients. Our findings have potential clinical implications in patient stratification and tailored therapy. 

## 4. Materials and Methods

### 4.1. Study Cohorts

RNA-Seq data were retrieved from the PRJNA688066 [14] (discovery) and GSE192341 [15] (validation cohort): Clinical characteristics for patients from both cohorts are shown in Table 1.

### 4.2. Data Retrieval and Bioinformatics

The Kallisto index was generated by creating a de Bruijn graph using the reference transcriptome GENCODE release (V33) and a *k*-mer length of 31. FASTQ files from the PRJNA688066 dataset were mapped and aligned to the generated GENCODE release (V33) index using Kallisto v0.46 as described before [18,19]. Expression values (TPM) were subjected to differential, principal component, and clustering analysis in AltAnalyze as described before [20,21]. Expression data from the validation cohort was retrieved from the gene omnibus (GEO) dataset GSE192341. 

### 4.3. Statistical Analysis

ROC analysis was employed to identify gene sets predictive of RD and pCR using SPSS version 26. The volcano plot was generated in GraphPad prism (v9.0) by plotting the AUC on the *y*-axis and −log10 *p*-value on the *x*-axis. Other plots were also generated in GraphPad prism v9.0. 

### 4.4. Ingenuity Pathway Analysis

Differentially expressed genes in RD vs. pCR were imported into the IPA software (Ingenuity Systems; www.ingenuity.com/ accessed on 4 August 2022).) and were used for functional annotations and network analysis using canonical, upstream regulator, and downstream effector analyses The *p*-value is the negative log of P and represents the possibility that focus genes in the network being found together by chance [7,22]. 

### 4.5. Discriminant Analyses

To determine the ability of predicted variables (genes identified from the ROC analysis) to discriminate between RD and pCR, discriminant analyses were performed using an OPLS-DA classifier using Soft Independent Modelling by Class Analogy (SIMCA) software (version 16; Umetrics, Sweden) on the discovery dataset (*n* = 90). The model was then tested on the validation dataset (*n* = 50) and the performance was assessed by generating ROC curve and determining the AUC value. The sensitivity and specificity constants of the test were determined based on similar classification scores by OPLS-DA.

### 4.6. Protein–Protein Interaction (PPI)

PPI network analysis was conducted using the STRING database as described before [23]. The 500 identified common genes or the 87-immune-gene signatures predictive of pCR were subjected to PPI in the STRING database v 11.5 (https://string-db.org/ accessed on 4 August 2022) as described before [24,25]. 

### 4.7. CRISPR-Cas9 Screen Data Retrieval

Genome-wide CRISPR-Cas9 screen data were retrieved from the Achilles project and gene effect score of ≤−0.5 for TNBC cell lines were included in the analysis [8]. 

### 4.8. Gene Silencing Using SiRNA

The scrambled siRNA control and ON-TARGETplus SMARTpool siRNA targeting human ELOB, SLC39A7, TIMM13, BANF1, NDUFS1, NDUFB7, TRAPPC5, and MVD were obtained from Dharmacon (Lafayette, CO, USA). Transfection was carried out using a reverse transfection strategy as previously described [19]. Briefly, siRNA was diluted in 50 μL of Opti-MEM (GIBCO, Carlsbad, CA, USA), and 1 μL of Lipofectamine 2000 (Invitrogen) was diluted in 50 μL of OPTI-MEM. The diluted siRNA (at a final concentration of 30 nM) and Lipofectamine 2000 were mixed together and allowed to form complexes at room temperature for 20 min. For transfection, twenty microliters of transfection mixture were first placed in individual wells of the tissue culture plate, and then 10,000 of MDA-MB-231 or BT-549 TNBC cells in 60 μL transfection medium (complete DMEM without Pen-Strep) were added to each well. The transfection cocktail was then replaced with complete DMEM one day later.

### 4.9. Colony Forming Unity (CFU) Assay and Chemotherapy Sensitivity of TNBC Models

Transfected cells were cultured for 48 h and were then exposed to paclitaxel (10 nM). Three days later, cells were fixed for 5 min using 4% PFA and then were washed twice using PBS followed by staining with crystal violet (0.1% in 10% EtOH) at room temperature for 10 min. The images were then captured and compared to experiment controls. The plates were then allowed to air dry, followed by CFU quantification of dissolved crystal violet in 5% SDS. Absorbance was measured at 590 nm. The experiments were repeated at least twice, and data were presented as the mean ± SD from four replicas.

### 4.10. Detection of Cell Death Using Fluorescence Microscopy

AO/EtBr fluorescence microscopy was used to assess apoptotic cells in TNBC cells after gene knockdown as single treatment or in combination with paclitaxel in TNBC cells, as we previously described [26]. Briefly, TNBC cells were transfected in a 24-well flat-bottom tissue culture plate. Forty eight hours later, media was changed and paclitaxel was added at a final concentration of 7.5 nM. Two days later, TNBC cells were washed twice using PBS and were subsequently stained with dual fluorescent staining solution (100 μg/mL AO and 100 μg/mL EtBr) for 2 min. AO and EtBr were purchased from Sigma (Sigma Aldrich, St. Louis, MO, USA);Stained ells were then visualized under inverted microscope (Olympus IX73 fluorescence microscope, Olympus, Tokyo, Japan). AO staining was used to visualize the number of cells that had undergone apoptosis, while EtBr-positive cells indicated necrotic cells or late apoptotic cell, with the later also showing condensed chromatin.

### 4.11. Survival Analysis

Gene signatures predictive of RD or pCR were subjected to Kaplan–Meier survival analysis (mean expression) using the KMplotter database (https://kmplot.com/ accessed on 4 August 2022) in breast cancer disease cohorts of basal (RFS: *n* = 442, OS: *n* = 296, DMFS: *n* = 283), HER2+ (RFS: *n* = 358, OS: *n* = 198, DMFS: *n* = 218), Luminal B (RFS: *n* = 566, OS: *n* = 200, DMFS: *n* = 183), and Luminal A (RFS: *n* = 631, OS: *n* = 222, DMFS: *n* = 259) as described before [27]. 

## 5. Conclusions

Our data have identified gene signatures predicative of RD and pCR in TNBC with potential clinical implications. 

## Figures and Tables

**Table 1:** Characteristic features of study population in the discovery and validation cohorts.

**Molecular Subtype** TNBC	**Discovery** **90** (100%)	**Validation** **50** (100%)
**Gender** Female	**90** (100%)	**50** (100%)
**Age (Median)**	**23–74** (53)	**26–73** (44) *
**Stage** NA I II III	**2** (2.2%) **12** (13.3%) **54** (60%) **22** (24.5%)	**9** (18.0%) **4** (8.0%) **29** (58.0%) **8** (16.0%)
**NAC** Taxane-based chemotherapy	**90** (100%)	**50** (100%) **
**Response to NAC** RD pCR	**38** (42.2%) **52** (57.8%)	**29** (58.0%) **21** (52.0%)

NAC: neoadjuvant chemotherapy; RD: residual disease; pCR: pathological complete response. * Age information for 9 patients not available. ** Type of chemotherapy not indicated.

## Data Availability

Data are available as Supplementary Materials. Source data and accession numbers are indicated in Section 4.

## Supplementary Materials

The following supporting information can be downloaded at: https://www.mdpi.com/article/10.3390/ijms231810901/s1.
