# Supplementary material for: Identification of a Gene Panel Predictive of Triple-Negative Breast Cancer Response to Neoadjuvant Chemotherapy Employing Transcriptomic and Functional Validation
Source: Int J Mol Sci. 2022 Sep 17;23(18):10901. doi: 10.3390/ijms231810901 (PMC9506546; doi:10.3390/ijms231810901)
Supplement: Supplementary file 1 [file ijms-23-10901-s001.zip › Figure S1.pdf]

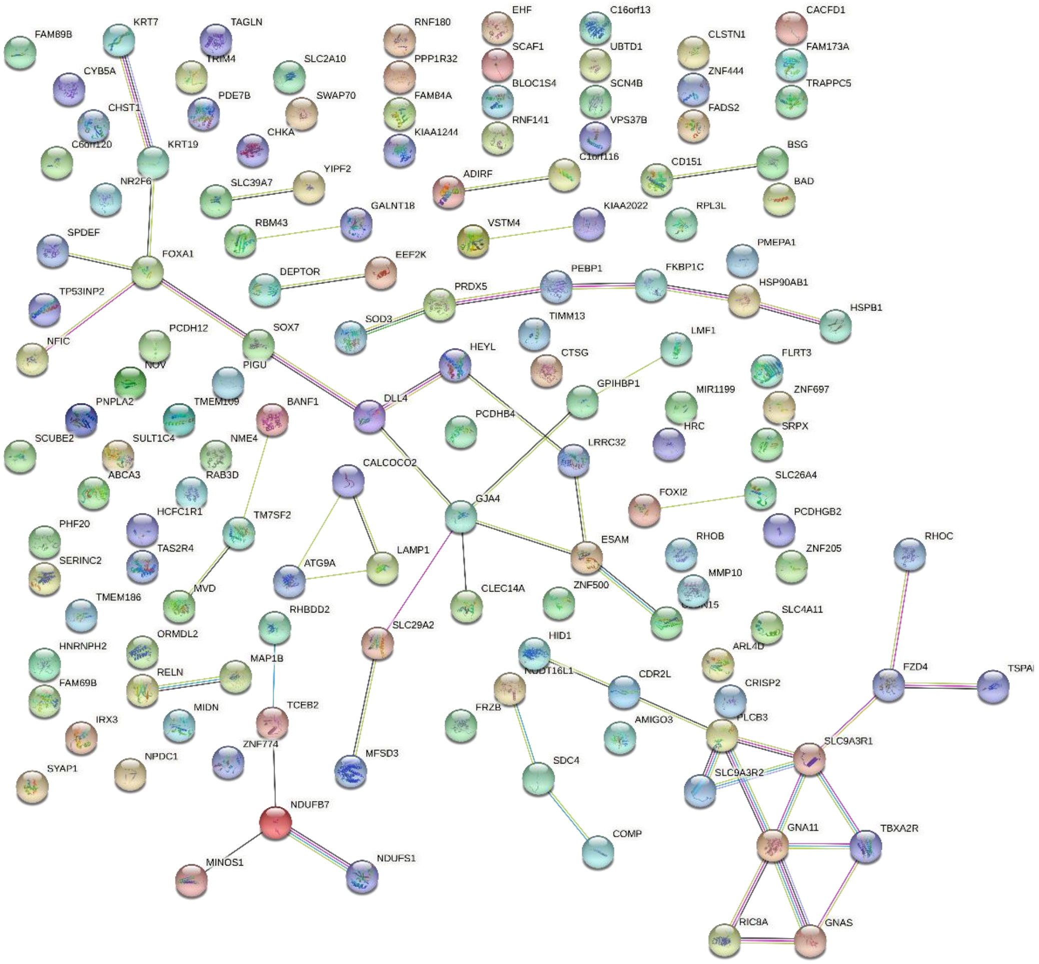


**Figure S1. PPI network on 140 genes predictive of RD.** Network statistics: number of nodes: 139; number of edges: 57; average node degree: 0.82; avg. local clustering coefficient: 0.305; expected number of edges: 43; PPI enrichment p-value: 0.0276.
