# Supplementary material for: Identification of a Gene Panel Predictive of Triple-Negative Breast Cancer Response to Neoadjuvant Chemotherapy Employing Transcriptomic and Functional Validation
Source: Int J Mol Sci. 2022 Sep 17;23(18):10901. doi: 10.3390/ijms231810901 (PMC9506546; doi:10.3390/ijms231810901)
Supplement: Supplementary file 1 [file ijms-23-10901-s001.zip › Figure S2.pdf]

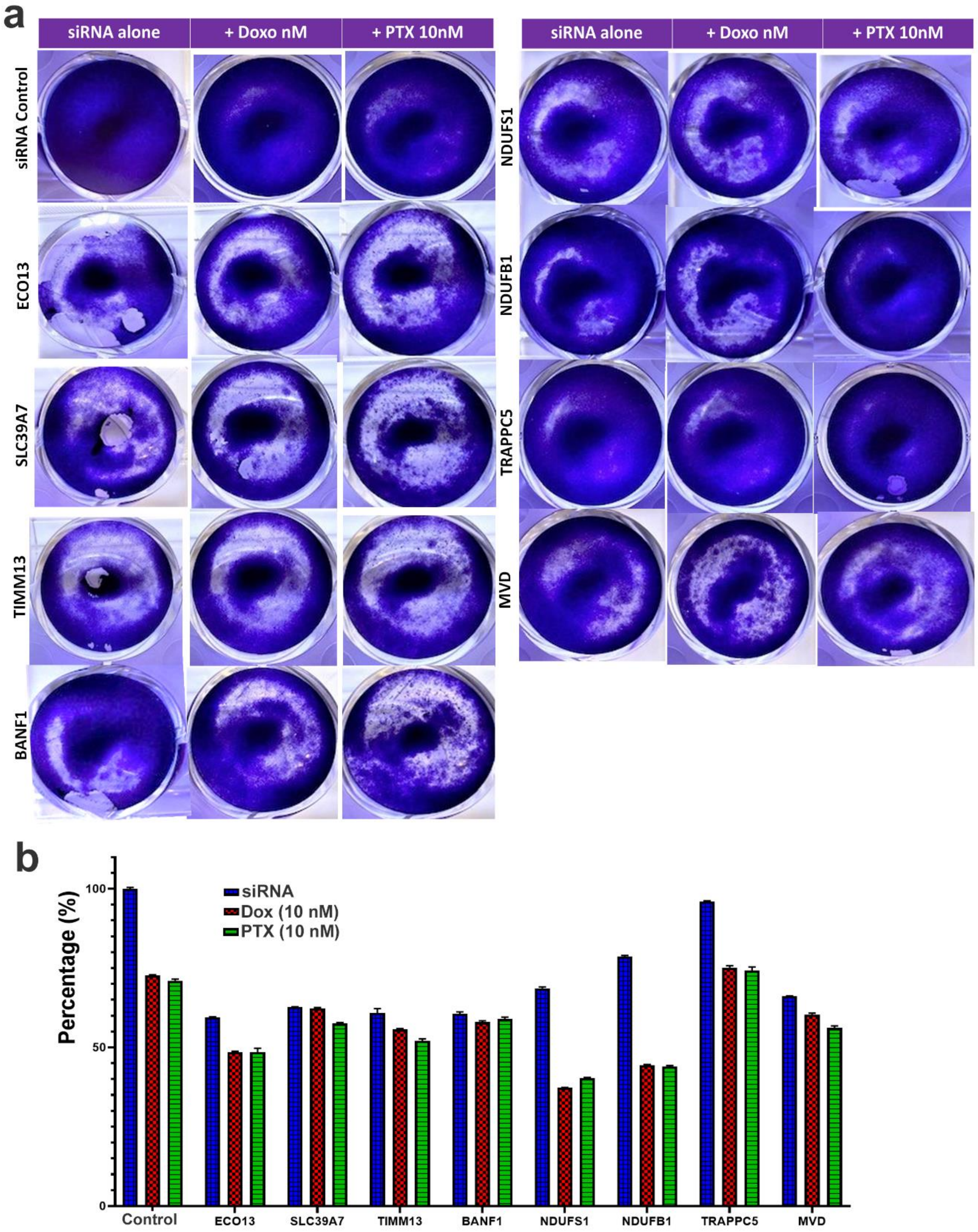

Figure S2. CFU of BT-549 in reponse to siRNA gene knockdown. (a) BT-549 were treated with the indicated siRNA as single agent or in combiantion (after 48 hrs) with doxrubcin (10 nM) or Paclitaxel (10 nM) and were stained with cystal violet on day 5 post transfection. **(b)** Quantification of crytal violet from a using spectrophotometry.
