# Supplementary material for: Identification of a Gene Panel Predictive of Triple-Negative Breast Cancer Response to Neoadjuvant Chemotherapy Employing Transcriptomic and Functional Validation
Source: Int J Mol Sci. 2022 Sep 17;23(18):10901. doi: 10.3390/ijms231810901 (PMC9506546; doi:10.3390/ijms231810901)
Supplement: Supplementary file 1 [file ijms-23-10901-s001.zip › Figure S3.pdf]

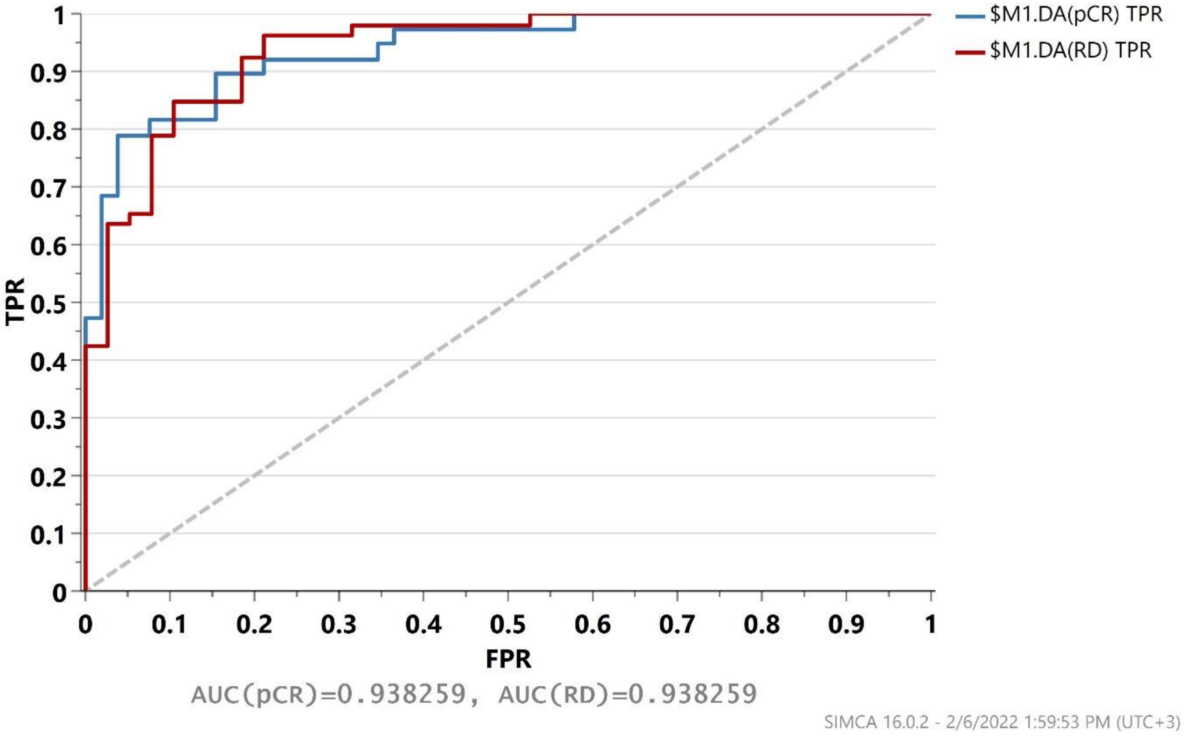

Figure S3. ROC analysis of predictive gene signature on the discovery cohort. ROC analysis of the identified gene signature predictive of RD and pCR in the discovery cohort consisting of 38 RD and 52 pCR. Y-axis represent sensitivity while the x-axis represent 1-specificity.
